# Diaqua­bis[5-(5-carboxy-2-pyridyl)tetra­zolato-κ^2^ *N* ^1^,*N* ^5^]cadmium(II) dihydrate

**DOI:** 10.1107/S1600536809007399

**Published:** 2009-03-11

**Authors:** Haoyong Yin, Ling Wang, Qiulin Nie

**Affiliations:** a Institute of Environmental Science and Engineering, Hangzhou Dianzi University, Hangzhou, 310018, People’s Republic of China; b College of Chemistry and Chemical Engineering, Xinyang Normal University, Xinyang, 464000, People’s Republic of China

## Abstract

In the title complex, [Cd(C_7_H_4_N_5_O_2_)_2_(H_2_O)_2_]·2H_2_O, the water-coordinated Cd^II^ atom (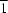 symmetry) is coordinated by four N atoms from two symmetry-related 3-carboxy­pyidyl-6-tetra­zolato ligands, forming a distorted octa­hedral complex. The uncoordinated water mol­ecules connect the mononuclear units into a layer structure through O—H⋯N and O—H⋯O hydrogen bonds; similar hydrogen bonds between coordinated water mol­ecules and anionic groups result in a three-dimensional structure.

## Related literature

For background, see: Xiong *et al.* (2002 ▶)
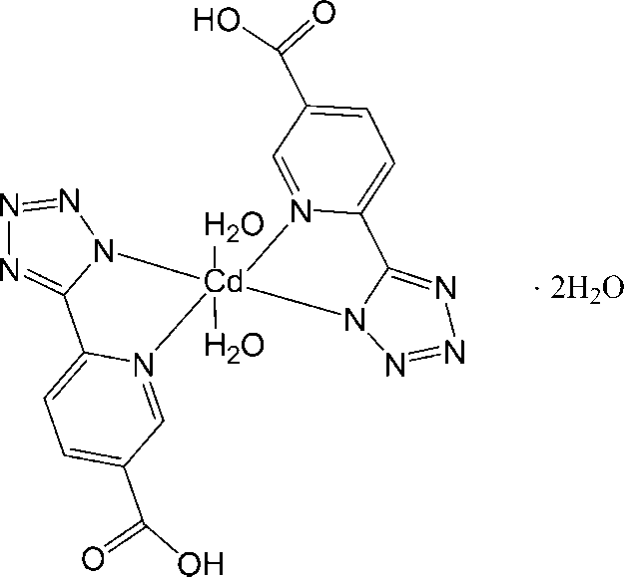


## Experimental

### 

#### Crystal data

[Cd(C_7_H_4_N_5_O_2_)_2_(H_2_O)_2_]·2H_2_O
*M* *_r_* = 564.77
Triclinic, 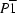

*a* = 6.1018 (2) Å
*b* = 7.3805 (1) Å
*c* = 12.383 (2) Å
α = 84.17 (3)°
β = 88.91 (3)°
γ = 65.71 (2)°
*V* = 505.51 (8) Å^3^
*Z* = 1
Mo *K*α radiation
μ = 1.15 mm^−1^
*T* = 293 K
0.20 × 0.20 × 0.20 mm

#### Data collection

Rigaku Mercury CCD diffractometer
Absorption correction: multi-scan (*CrystalClear*; Rigaku, 2000 ▶) *T*_min_ = 0.773, *T*_max_ = 1.000 (expected range = 0.615–0.795)
3817 measured reflections
2286 independent reflections
2104 reflections with *I* > 2σ(*I*)
*R*_int_ = 0.027

#### Refinement

*R*[*F* ^2^ > 2σ(*F* ^2^)] = 0.037
*wR*(*F* ^2^) = 0.078
*S* = 1.10
2286 reflections
171 parameters
5 restraints
H atoms treated by a mixture of independent and constrained refinement
Δρ_max_ = 0.39 e Å^−3^
Δρ_min_ = −0.70 e Å^−3^

### 

Data collection: *CrystalClear* (Rigaku, 2000 ▶); cell refinement: *CrystalClear*; data reduction: *CrystalClear*; program(s) used to solve structure: *SHELXS97* (Sheldrick, 2008 ▶); program(s) used to refine structure: *SHELXL97* (Sheldrick, 2008 ▶); molecular graphics: *X-SEED* (Barbour, 2001 ▶); software used to prepare material for publication: *SHELXL97*.

## Supplementary Material

Crystal structure: contains datablocks I, global. DOI: 10.1107/S1600536809007399/ng2554sup1.cif

Structure factors: contains datablocks I. DOI: 10.1107/S1600536809007399/ng2554Isup2.hkl

Additional supplementary materials:  crystallographic information; 3D view; checkCIF report

## Figures and Tables

**Table 1:** Selected geometric parameters (Å, °)

Cd1—N5	2.293 (2)
Cd1—O3	2.312 (3)
Cd1—N1	2.396 (2)

Symmetry code: (i) 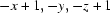.

**Table 2:** Hydrogen-bond geometry (Å, °)

*D*—H⋯*A*	*D*—H	H⋯*A*	*D*⋯*A*	*D*—H⋯*A*
O4—H4*B*⋯N2	0.85 (3)	2.02 (3)	2.860 (4)	166 (4)
O4—H4*A*⋯O2^ii^	0.85 (3)	1.92 (3)	2.767 (4)	170 (4)
O1—H1⋯O4^iii^	0.89 (3)	1.68 (3)	2.566 (4)	170 (5)
O3—H3*B*⋯N4^iv^	0.86 (2)	1.96 (3)	2.804 (4)	168 (4)
O3—H3*A*⋯N3^v^	0.90 (2)	1.92 (2)	2.806 (4)	172 (3)

Symmetry codes: (ii) 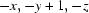; (iii) 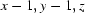; (iv) 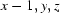; (v) 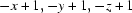.

**Fig. 1.**
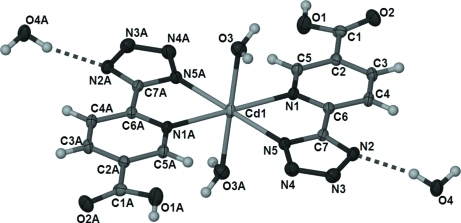
ORTEP of complex (I) with 30% thermal ellipsoids. A = 1 - x, -y, 1 - z

**Fig. 2.**
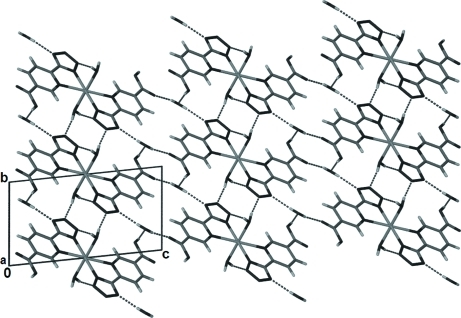
The packing structure viewed along a axis.

## supplementary crystallographic information

### Comment

Hydrothermal reactions involving *in situ* ligand synthesis have attracted great interests (Xiong *et al.*, 2002). In the contribution, we report the title mononuclear complex (I) based on tetrazol ligand obtained by *in situ* ligand synthesis.

In the structure of (I), the ligand chelates Cd(II) center through pyridyl N and tetrazol N to form a centrosymmetrical mononuclear complex. Two coordinated water molecules complete the octahedral geometry of Cd(II) center (Fig.1). Two solvent water molecules and carboxylic groups of the ligands form a synthon *R*_4_^4^(12) which connects mononuclear unit into a two-dimensional layer structure through hydrogen bonds between solvent water and tetrazol groups (Table. 2). The hydrogen bonds between coordinated water molecules and tetrazol groups result in a three-dimensional structure (Fig.2).

### Experimental

A mixture of Cd(NO_3_)_2_.4H_2_O (77 mg, 0.25 mmol), sodium azide(33 mg, 0.5 mmol) and 6-cyanopyridine-3-carboxylic acid (74 mg, 0.5 mmol) was suspended in water (10 ml) and heated in a teflon-lined steel bomb at 160 ° C for 3 days. The colorless crystals were obtained.

### Refinement

H atoms bonded to C were located geometrically (C—H = 0.95 Å) with *U*_iso_(H) = 1.2 *U*_eq_(C). H atoms bonded to O were located by difference maps and refined with a distance restraint of O—H = 0.87 (3) Å. The displacement factors were freely refined.

### Crystal data

**Table:**

[Cd(C_7_H_4_N_5_O_2_)_2_(H_2_O)_2_]·2H_2_O	*Z* = 1
*M**_r_* = 564.77	*F*(000) = 282
Triclinic, *P*1	*D*_x_ = 1.855 Mg m^−^^3^
Hall symbol: -P 1	Mo *K*α radiation, λ = 0.71073 Å
*a* = 6.1018 (2) Å	Cell parameters from 612 reflections
*b* = 7.3805 (1) Å	θ = 3.0–27.5°
*c* = 12.383 (2) Å	µ = 1.15 mm^−^^1^
α = 84.17 (3)°	*T* = 293 K
β = 88.91 (3)°	Prism, colorless
γ = 65.71 (2)°	0.20 × 0.20 × 0.20 mm
*V* = 505.51 (8) Å^3^	

### Data collection

**Table:**

Rigaku Mercury CCD diffractometer	2286 independent reflections
Radiation source: fine-focus sealed tube	2104 reflections with *I* > 2σ(*I*)
graphite	*R*_int_ = 0.027
Detector resolution: 13.6612 pixels mm^-1^	θ_max_ = 27.4°, θ_min_ = 3.0°
CCD_Profile_fitting scans	*h* = −7→7
Absorption correction: multi-scan (*CrystalClear*; Rigaku, 2000)	*k* = −7→9
*T*_min_ = 0.773, *T*_max_ = 1.000	*l* = −15→15
3817 measured reflections	

### Refinement

**Table:**

Refinement on *F*^2^	Primary atom site location: structure-invariant direct methods
Least-squares matrix: full	Secondary atom site location: difference Fourier map
*R*[*F*^2^ > 2σ(*F*^2^)] = 0.037	Hydrogen site location: inferred from neighbouring sites
*wR*(*F*^2^) = 0.078	H atoms treated by a mixture of independent and constrained refinement
*S* = 1.10	*w* = 1/[σ^2^(*F*_o_^2^) + (0.0287*P*)^2^ + 0.1909*P*] where *P* = (*F*_o_^2^ + 2*F*_c_^2^)/3
2286 reflections	(Δ/σ)_max_ = 0.001
171 parameters	Δρ_max_ = 0.39 e Å^−^^3^
5 restraints	Δρ_min_ = −0.70 e Å^−^^3^

### Special details

**Table:**

Geometry. All e.s.d.'s (except the e.s.d. in the dihedral angle between two l.s. planes) are estimated using the full covariance matrix. The cell e.s.d.'s are taken into account individually in the estimation of e.s.d.'s in distances, angles and torsion angles; correlations between e.s.d.'s in cell parameters are only used when they are defined by crystal symmetry. An approximate (isotropic) treatment of cell e.s.d.'s is used for estimating e.s.d.'s involving l.s. planes.
Refinement. Refinement of *F*^2^ against ALL reflections. The weighted *R*-factor *wR* and goodness of fit *S* are based on *F*^2^, conventional *R*-factors *R* are based on *F*, with *F* set to zero for negative *F*^2^. The threshold expression of *F*^2^ > σ(*F*^2^) is used only for calculating *R*-factors(gt) *etc*. and is not relevant to the choice of reflections for refinement. *R*-factors based on *F*^2^ are statistically about twice as large as those based on *F*, and *R*- factors based on ALL data will be even larger.

### Fractional atomic coordinates and isotropic or equivalent isotropic displacement parameters (Å^2^)

**Table:**

	*x*	*y*	*z*	*U*_iso_*/*U*_eq_	
Cd1	0.5000	0.0000	0.5000	0.03880 (13)	
O1	−0.1058 (5)	−0.1234 (4)	0.1759 (2)	0.0614 (8)	
N1	0.3050 (5)	0.1080 (4)	0.32445 (19)	0.0328 (6)	
C1	−0.1196 (6)	0.0465 (5)	0.1283 (3)	0.0401 (7)	
H1	−0.217 (7)	−0.160 (7)	0.152 (4)	0.098 (17)*	
O2	−0.2500 (5)	0.1386 (4)	0.0504 (2)	0.0580 (7)	
N2	0.5549 (5)	0.4825 (4)	0.2873 (2)	0.0404 (6)	
C2	0.0443 (6)	0.1228 (4)	0.1777 (2)	0.0338 (7)	
O3	0.1539 (5)	0.2229 (4)	0.5726 (2)	0.0512 (6)	
N3	0.7080 (5)	0.4857 (4)	0.3617 (2)	0.0461 (7)	
C3	0.0743 (6)	0.2851 (5)	0.1257 (3)	0.0425 (8)	
H3	−0.0060	0.3468	0.0581	0.051*	
H3A	0.195 (6)	0.321 (4)	0.587 (2)	0.031 (8)*	
H3B	0.015 (5)	0.273 (5)	0.540 (3)	0.052 (11)*	
O4	0.6141 (5)	0.7305 (4)	0.1064 (2)	0.0579 (7)	
N4	0.7355 (5)	0.3463 (4)	0.4431 (2)	0.0450 (7)	
C4	0.2210 (6)	0.3572 (5)	0.1722 (3)	0.0424 (8)	
H4	0.2453	0.4676	0.1367	0.051*	
H4A	0.492 (6)	0.781 (6)	0.063 (3)	0.062 (12)*	
H4B	0.571 (7)	0.666 (6)	0.157 (3)	0.068 (13)*	
N5	0.6029 (5)	0.2491 (4)	0.4230 (2)	0.0354 (6)	
C5	0.1627 (6)	0.0381 (5)	0.2777 (2)	0.0366 (7)	
H5	0.1418	−0.0732	0.3141	0.044*	
C6	0.3338 (5)	0.2658 (4)	0.2722 (2)	0.0326 (6)	
C7	0.4943 (6)	0.3343 (4)	0.3263 (2)	0.0340 (7)	

### Atomic displacement parameters (Å^2^)

**Table:**

	*U*^11^	*U*^22^	*U*^33^	*U*^12^	*U*^13^	*U*^23^
Cd1	0.0443 (2)	0.0424 (2)	0.03052 (19)	−0.02092 (16)	−0.00857 (14)	0.00836 (13)
O1	0.075 (2)	0.0752 (19)	0.0503 (16)	−0.0506 (17)	−0.0210 (14)	0.0103 (13)
N1	0.0369 (15)	0.0352 (13)	0.0274 (13)	−0.0169 (12)	0.0003 (10)	0.0014 (10)
C1	0.0366 (19)	0.054 (2)	0.0307 (16)	−0.0186 (16)	0.0024 (13)	−0.0080 (14)
O2	0.0524 (17)	0.0714 (17)	0.0467 (15)	−0.0237 (14)	−0.0215 (12)	0.0051 (12)
N2	0.0446 (17)	0.0393 (15)	0.0422 (16)	−0.0237 (13)	−0.0003 (12)	0.0031 (11)
C2	0.0324 (17)	0.0403 (16)	0.0252 (15)	−0.0118 (13)	−0.0002 (12)	−0.0016 (12)
O3	0.0495 (18)	0.0497 (15)	0.0547 (16)	−0.0199 (14)	−0.0028 (13)	−0.0076 (12)
N3	0.0491 (19)	0.0470 (16)	0.0503 (18)	−0.0276 (15)	0.0015 (14)	−0.0062 (13)
C3	0.042 (2)	0.0482 (19)	0.0329 (17)	−0.0168 (16)	−0.0089 (14)	0.0099 (14)
O4	0.0610 (19)	0.0764 (19)	0.0474 (16)	−0.0446 (16)	−0.0197 (14)	0.0201 (14)
N4	0.0446 (18)	0.0470 (16)	0.0478 (17)	−0.0231 (14)	−0.0064 (13)	−0.0032 (13)
C4	0.046 (2)	0.0419 (18)	0.0370 (18)	−0.0190 (16)	−0.0024 (14)	0.0105 (13)
N5	0.0352 (15)	0.0353 (13)	0.0381 (14)	−0.0178 (12)	−0.0034 (11)	0.0014 (11)
C5	0.0393 (19)	0.0405 (17)	0.0315 (16)	−0.0194 (15)	−0.0029 (13)	0.0032 (12)
C6	0.0321 (17)	0.0321 (15)	0.0312 (16)	−0.0114 (13)	0.0011 (12)	0.0000 (12)
C7	0.0345 (17)	0.0319 (15)	0.0336 (16)	−0.0128 (13)	0.0034 (12)	0.0017 (12)

### Geometric parameters (Å, °)

**Table:**

Cd1—N5	2.293 (2)	C2—C5	1.398 (4)
Cd1—N5^i^	2.293 (2)	O3—H3A	0.90 (2)
Cd1—O3^i^	2.312 (3)	O3—H3B	0.86 (2)
Cd1—O3	2.312 (3)	N3—N4	1.325 (4)
Cd1—N1	2.396 (2)	C3—C4	1.374 (5)
Cd1—N1^i^	2.396 (2)	C3—H3	0.9500
O1—C1	1.301 (4)	O4—H4A	0.85 (3)
O1—H1	0.89 (3)	O4—H4B	0.85 (3)
N1—C5	1.342 (4)	N4—N5	1.324 (4)
N1—C6	1.348 (4)	C4—C6	1.394 (4)
C1—O2	1.215 (4)	C4—H4	0.9500
C1—C2	1.499 (4)	N5—C7	1.343 (4)
N2—N3	1.332 (4)	C5—H5	0.9500
N2—C7	1.336 (4)	C6—C7	1.472 (4)
C2—C3	1.379 (4)		
			
N5—Cd1—N5^i^	180.0	C5—C2—C1	121.8 (3)
N5—Cd1—O3^i^	87.03 (10)	Cd1—O3—H3A	103 (2)
N5^i^—Cd1—O3^i^	92.97 (10)	Cd1—O3—H3B	124 (3)
N5—Cd1—O3	92.97 (10)	H3A—O3—H3B	109 (3)
N5^i^—Cd1—O3	87.03 (10)	N4—N3—N2	109.7 (3)
O3^i^—Cd1—O3	180.0	C4—C3—C2	119.7 (3)
N5—Cd1—N1	72.97 (9)	C4—C3—H3	120.2
N5^i^—Cd1—N1	107.03 (9)	C2—C3—H3	120.2
O3^i^—Cd1—N1	91.55 (9)	H4A—O4—H4B	103 (4)
O3—Cd1—N1	88.45 (9)	N5—N4—N3	109.3 (3)
N5—Cd1—N1^i^	107.03 (9)	C3—C4—C6	119.0 (3)
N5^i^—Cd1—N1^i^	72.97 (9)	C3—C4—H4	120.5
O3^i^—Cd1—N1^i^	88.45 (9)	C6—C4—H4	120.5
O3—Cd1—N1^i^	91.55 (9)	N4—N5—C7	105.1 (2)
N1—Cd1—N1^i^	180.00 (5)	N4—N5—Cd1	140.8 (2)
C1—O1—H1	113 (3)	C7—N5—Cd1	114.08 (19)
C5—N1—C6	118.3 (2)	N1—C5—C2	122.5 (3)
C5—N1—Cd1	127.62 (19)	N1—C5—H5	118.7
C6—N1—Cd1	113.95 (18)	C2—C5—H5	118.7
O2—C1—O1	124.7 (3)	N1—C6—C4	122.1 (3)
O2—C1—C2	121.4 (3)	N1—C6—C7	116.0 (2)
O1—C1—C2	113.9 (3)	C4—C6—C7	121.9 (3)
N3—N2—C7	104.7 (3)	N2—C7—N5	111.3 (3)
C3—C2—C5	118.4 (3)	N2—C7—C6	126.0 (3)
C3—C2—C1	119.8 (3)	N5—C7—C6	122.7 (3)

Symmetry codes: (i) −*x*+1, −*y*, −*z*+1.

### Hydrogen-bond geometry (Å, °)

**Table:**

*D*—H···*A*	*D*—H	H···*A*	*D*···*A*	*D*—H···*A*
O4—H4B···N2	0.85 (3)	2.02 (3)	2.860 (4)	166 (4)
O4—H4A···O2^ii^	0.85 (3)	1.92 (3)	2.767 (4)	170 (4)
O1—H1···O4^iii^	0.89 (3)	1.68 (3)	2.566 (4)	170 (5)
O3—H3B···N4^iv^	0.86 (2)	1.96 (3)	2.804 (4)	168 (4)
O3—H3A···N3^v^	0.90 (2)	1.92 (2)	2.806 (4)	172 (3)

Symmetry codes: (ii) −*x*, −*y*+1, −*z*; (iii) *x*−1, *y*−1, *z*; (iv) *x*−1, *y*, *z*; (v) −*x*+1, −*y*+1, −*z*+1.
